# Soluble TIM-3 and galectin-9 in cerebrospinal fluid are associated with disability at 6 months in aquaporin-4 IgG-positive neuromyelitis optica spectrum disorder

**DOI:** 10.3389/fimmu.2026.1923404

**Published:** 2026-10-05

**Authors:** Shota Nishii, Ritsu Akatani, Kyoka Shiroma, Ryosuke Takeda, Asato Tsuji, Kimitaka Katanazaka, Shusuke Koto, Norio Chihara

**Affiliations:** Division of Neurology, Kobe University Graduate School of Medicine, Kobe, Japan

**Keywords:** biomarker, CSF, galectin 9 (Gal-9), neuromyelitis optica (NMO), TIM-3

## Abstract

**Background:**

Neuromyelitis optica spectrum disorders (NMOSD) are severe autoimmune diseases of the central nervous system characterized by autoantibodies against aquaporin 4 (AQP4-IgG). Despite the availability of several molecular targeted therapies, biomarkers capable of predicting subsequent disability early during the acute relapse phase remain insufficient. Given that abnormalities in immune checkpoint molecules are known to be involved in autoimmunity, this study investigated whether immune checkpoint-related molecules in the cerebrospinal fluid (CSF) of acute-phase AQP4-IgG-positive NMOSD patients correlate with disability at 6 months.

**Methods:**

Thirty-four patients with AQP4 IgG-positive NMOSD, 10 patients with myelin oligodendrocyte glycoprotein antibody-associated disease (MOGAD)at disease flare, and 11 patients with idiopathic normal pressure hydrocephalus (iNPH) as control non-inflammatory neurological disease were enrolled. Bead-based multiplex assays were used to quantify levels of immune checkpoint molecules and related factors (PD-1, PD-L1, CTLA-4, TIM-3, galectin-9, LAG-3, 4-1BB, B7.2, IL-2Rα, TGF-β1, FasL) in CSF. These molecules were measured in the soluble fraction. Disease severity at 6 months was classified as mild (EDSS < 6) or severe (EDSS ≥ 6). ROC analysis was performed to evaluate prognostic performance.

**Results:**

Twenty-six cases of NMOSD showed mild disability, and 8 cases showed severe disability. All cases of MOGAD showed mild disability. Compared to the mild group, the severe group had higher age, a higher male ratio, and higher cerebrospinal fluid protein levels (p=0.004, p=0.017, p<0.001, respectively). Cerebrospinal fluid levels of TIM-3, galectin-9, and IL-2Rα were significantly higher in the severe group (p=0.011, p=0.008, p=0.006, respectively), with area under the ROC curve values of 0.89, 0.88, and 0.80, respectively.

**Conclusion:**

In AQP4-IgG-positive NMOSD patients, elevated levels of TIM-3, galectin-9, and IL-2Rα in CSF during the acute attack phase correlate with worsening disability severity at 6 months. These findings suggest their potential as early prognostic biomarkers, which requires validation in a large cohort.

## Introduction

Neuromyelitis optica spectrum disorders (NMOSD) is a spectrum of autoimmune diseases in the central nervous system (CNS) primarily affecting the optic nerves and spinal cord. NMOSD are characterized by the autoantibodies against aquaporin 4 (AQP4-IgG) which contribute to the pathogenesis. Although prognosis has improved with the advent of targeted therapies, its prognosis remains poor (1). To improve prognosis, early treatment and prevention of recurrence tailored to the severity is therefore crucial. Although several molecular targeted therapies are now available, no biomarkers have been established to predict the severity in the acute phase of NMOSD. Consequently, reliable prognostic biomarkers for NMOSD are required. Myelin oligodendrocyte glycoprotein antibody-associated disease (MOGAD) is an emerging autoimmune disease in the CNS associated with autoantibody against myelin oligodendrocyte glycoprotein which shows comparable clinical phenotype with NMOSD.

Immune checkpoint molecules regulate autoimmune T cells in CNS (2). Previous studies have investigated whether immune checkpoint molecules are associated with the progression in NMOSD. One study reported that serum programmed cell death protein 1 (PD-1)/programmed cell death ligand 1 (PD-L1) levels were correlated with NMOSD relapse (3), while another study reported a negative correlation between serum monocyte lymphocyte activation gene 3 (LAG-3) expression and NMOSD severity (4). However, the usefulness of these prognostic predictors in NMOSD and MOGAD has not been fully established. CSF immune checkpoint molecules reflect neuroimmune regulation and may be associated with the severity of neuroimmunological diseases such as NMOSD and MOGAD.

This study aimed to determine whether CSF immune checkpoint molecules in neuroimmunological diseases are associated with future disability.

## Methods

### Study population

This is a retrospective cohort study that included patients treated at Kobe University between January 2015 and March 2025. Eligible patients were ≥ 18 years old and had CSF samples available for analysis. Thirty-four patients with NMOSD, 10 with MOGAD, and 11 with idiopathic normal pressure hydrocephalus (iNPH) were included. NMOSD and MOGAD were diagnosed in accordance with established diagnostic criteria (5, 6). iNPH was diagnosed as possible iNPH according to established guidelines (7). NMOSD patients were classified based on expanded disability status scale (EDSS) at 6 months as mild (EDSS < 6) or severe (EDSS ≥ 6) (8). CSF samples from patients with NMOSD and MOGAD were obtained within 3 months after relapse onset. CSF samples were acquired by lumbar puncture after informed consent was obtained.

### CSF analysis

We conducted a secondary analysis of data obtained from our previous study (9). While the primary study evaluated the levels of co-inhibitory proteins of patients with NMOSD, MOGAD for the differential diagnosis of primary CNS lymphoma, the current analysis focused on evaluate predicting disease severity of these inflammatory diseases of CNS from a different clinical perspective. Briefly, CSF samples were centrifuged immediately and stored at -80 °C. After thawing, samples were analyzed using a custom bead-based multiplex immune-assay kit (LEGENDplex™; BioLegend) (10). Only soluble proteins in centrifuged CSF supernatants were quantified. We simultaneously measured levels of PD-1, PD-L1, cytotoxic T-lymphocyte antigen 4 (CTLA-4), T-cell immunoglobulin and mucin domain 3 (TIM-3), galectin-9, LAG-3, transforming growth factor β (TGF-β), B7.2, 4-1BB, IL-2R**α** (interleukin-2 receptor chain alpha), and FasL (Fas ligand).

### Statistical analysis

To compare the proportions of categorical variables (such as sex) among the groups, we used Holm-adjusted pairwise Fisher’s exact test; for the comparison between mild NMOSD and severe NMOSD, we used Fisher’s exact test. In addition, to compare the distributions of continuous variables (such as age and levels of evaluated proteins) among the groups, we used Kruskal-Wallis test followed by Holm-adjusted Dunn’s test; for comparisons between mild NMOSD and severe NMOSD, we used Brunner-Munzel’s test. Receiver operating characteristic (ROC) curves were created, and the area under the curve (AUC) values were calculated to evaluate the prognostic accuracy of evaluated proteins. The threshold for significance was p<0.05. All statistical analyses were conducted using R version 4.4.1 with RStudio version 2022.07.2. Because this was a retrospective exploratory study using an existing cohort, no *a priori* sample calculation was performed.

## Results

### Clinical and CSF features of NMOSD patients

Demographic, clinical, and CSF characteristics were compared among the groups (NMOSD, MOGAD, and iNPH) as well as between mild and severe NMOSD and summarized in **Table 1** and **Table 2**. Age differed significantly among the groups; *post-hoc* analyses showed that age was highest for iNPH, followed by NMOSD and MOGAD (**Supplementary Table 1**). The patients with severe NMOSD were significantly older than those with mild NMOSD. Overall differences in gender were observed; *post-hoc* analyses showed that there were more women in NMOSD than MOGAD. Severe NMOSD had a higher male ratio than mild NMOSD. CSF leukocyte counts also differed significantly; *post-hoc* tests showed that NMOSD and MOGAD had higher CSF leukocyte counts than iNPH. The patients with severe NMOSD had higher CSF protein levels than mild NMOSD.

**Table 1:** Demographic and clinical characteristics in NMOSD, MOGAD, and iNPH patients.

Baseline characteristics	NMOSD (n=34)	MOGAD (n=10)	iNPH (n=11)	P-value
Age; years, median (range)	59 (21-91)	32 (19-62)	74 (71-82)	**<0.001**
Female; no. (%)	25 (74)	2 (20)	5 (45)	**0.007**
Smoking; no. (%)	10 (29)	3 (30)	1 (9)	0.42
Prior steroid exposure; no. (%)	9 (26)	2 (20)	0 (0)	0.17
Duration from initial onset; months, median (range)	2.5 (0.25-372)	0.75 (0.25-159)	24 (10-48)	0.11
Duration from latest episode onset; weeks, median (range)	2 (1-16)	1.5 (1-11)		
Pretreatment EDSS median (range)	3.5 (1.0-9.5)	3 (1.0-6.0)		
CSF leukocyte; (/uL) median (range)	5 (0-159)	3 (0-67)	0 (0-2)	**0.005**
CSF protein; (mg/dL) median (range)	46 (22-277)	43 (20-50)	38 (22-194)	0.27

Bold indicates significant difference among the groups (p<0.05).

CSF, cerebrospinal fluid; EDSS, expanded disability status scale; iNPH, idiopathic normal pressure hydrocephalus; MOGAD, myelin oligodendrocyte glycoprotein antibody-associated disease; NMOSD, neuromyelitis optica spectrum disorders.

**Table 2:** Demographic and clinical characteristics in mild and severe NMOSD patients.

Baseline characteristics	Mild NMOSD (n=26)	Severe NMOSD (n=8)	P-value
Age; years, median (range)	53 (21-78)	66 (52-91)	**0.004**
Female; no. (%)	22 (85)	3 (36)	**0.017**
Smoking; no. (%)	6 (23)	4 (50)	0.20
Prior steroid exposure; no. (%)	8 (31)	1 (13)	0.40
Duration from initial onset; months, median (range)	6 (0.25-372)	0.5 (0.25-300)	0.17
Duration from latest episode onset; weeks, median (range)	2 (1-16)	2 (1-6)	0.66
Pretreatment EDSS median (range)	3 (1.0-6.5)	8 (8-9.5)	**<0.001**
CSF leukocyte; (/uL) median (range)	4 (0-38)	11 (0-159)	0.11
CSF protein; (mg/dL) median (range)	43 (22-137)	69 (46-277)	**<0.001**

Bold indicates significant difference between mild and severe NMOSD (p<0.05).

CSF, cerebrospinal fluid; EDSS, expanded disability status scale; IQR, interquartile range; NMOSD, neuromyelitis optica spectrum disorders.

### CSF TIM-3 and galectin-9 were associated with poor outcome for NMOSD

CSF levels of TIM-3, galectin-9, IL-2Rα, LAG-3, B7.2, and FasL differed significantly among the groups (**Table 3**); *post-hoc* analyses showed CSF levels of TIM-3, galectin-9, and IL-2Rα were significantly higher in severe NMOSD compared with the other groups (**Supplementary Table 2**). Other markers showed limited discriminatory ability between severe NMOSD and the other groups. All markers demonstrated a diminished ability to differentiate between MOGAD and iNPH. ROC analysis showed good discriminatory performance for CSF TIM-3 and galectin-9 (**Figure 1**). CSF IL-2Rα showed moderate prognostic accuracy. Notably, three patients with pretreatment EDSS ≥ 6 achieved favorable functional outcomes (6-month EDSS < 6) and were therefore classified into the mild group. To determine whether CSF immune checkpoint markers could identify patients with favorable recovery despite severe disability at presentation, we performed an exploratory analysis limited to patients with pretreatment EDSS ≥ 6. CSF TIM-3 and galectin-9 levels were significantly lower in patients with favorable recovery than in those with persistent severe disability (**Supplementary Figure 1**).

**Table 3:** CSF markers compared among severe NMOSD, mild NMOSD, MOGAD, and iNPH.

Markers	Severe NMOSD (n=8)	Mild NMOSD (n=26)	MOGAD (n=10)	iNPH (n=11)	P-value
TIM-3	5631 (2935-12230)	1303 (813-2159)	1033 (638-1886)	811 (600-1278)	**<0.001**
Galectin-9	781 (551-1144)	172 (128-298)	209 (148-386)	142 (112-196)	**0.002**
IL-2Rα	91 (57-150)	0 (0-0)	0 (0-61)	0 (0-0)	**0.004**
PD-1	0 (0-0)	0 (0-0)	0 (0-0)	0 (0-0)	1.0
PD-L1	0.7 (0-3.4)	0 (0-1.4)	0 (0-0)	0 (0-0)	0.06
CTLA-4	0 (0-0)	0 (0-0)	0 (0-0)	0 (0-0)	0.45
LAG-3	0 (0-1000)	0 (0-0)	0 (0-0)	0 (0-0)	**0.026**
4-1BB	0 (0-0)	0 (0-0)	0 (0-0)	0 (0-0)	0.52
B7.2	26 (0-85)	0 (0-0)	0 (0-0)	0 (0-2.7)	**0.002**
FasL	5.1 (3.2-10.5)	2.7 (2.1-4.2)	3.1 (2.0-3.7)	1.4 (1.0-1.8)	**<0.001**
TGF-β	0 (0-0)	0 (0-0)	0 (0-0)	0 (0-0)	0.118

CSF markers were compared among severe NMOSD, mild NMOSD, MOGAD, and iNPH using Kruskal-Wallis test. Data are presented as medians (IQR) (pg/mL).

Bold indicates significant difference among the groups (p<0.05).

CSF, cerebrospinal fluid; CTLA-4, cytotoxic T-lymphocyte antigen 4; FasL, Fas ligand; IL-2Rα, interleukin-2 receptor subunit alpha; iNPH, idiopathic normal pressure hydrocephalus; IQR, interquartile range; LAG-3, lymphocyte activation gene 3; MOGAD, myelin oligodendrocyte glycoprotein antibody-associated disease; NMOSD, neuromyelitis optica spectrum disorders; PD-1, programmed cell death protein 1; PD-L1, programmed cell death ligand 1; TGF-β, transforming growth factor β; TIM-3, T-cell immunoglobulin and mucin domain 3.

**Figure 1:**
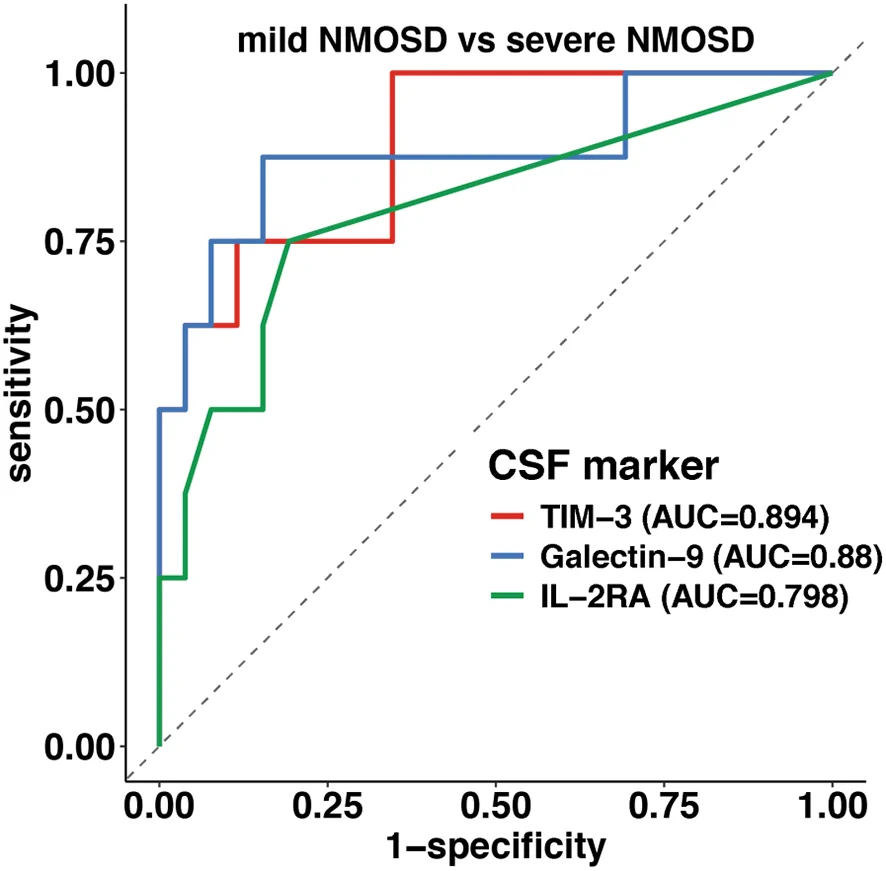
Accuracy of CSF prognostic markers for NMOSD. ROC curves showed the prognostic performance of CSF TIM-3, galectin-9, and IL-2Rα for distinguishing mild and severe NMOSD. The corresponding AUC values were 0.894 (95% CI, 0.772-1.0) for TIM-3, 0.880 (95% CI, 0.709-1.0) for galectin-9, and 0.798 (95% CI, 0.612-0.985) for IL-2Rα. AUC, area under the curve; CI, confidence interval; CSF, cerebrospinal fluid; IL-2Rα, interleukin-2 receptor chain alpha; NMOSD, neuromyelitis optica spectrum disorders; ROC, receiver operating characteristic; TIM-3, T-cell immunoglobulin and mucin domain 3.

### CSF TIM-3 and galectin-9 were correlated in NMOSD

Next, we studied the correlations between the CSF marker levels in NMOSD patients. Correlation analysis revealed TIM-3 and galectin-9 showed strong positive correlations with each other (**Figure 2**). On the other hand, correlations between IL-2Rα and TIM-3 or galectin-9 were moderate.

**Figure 2:**
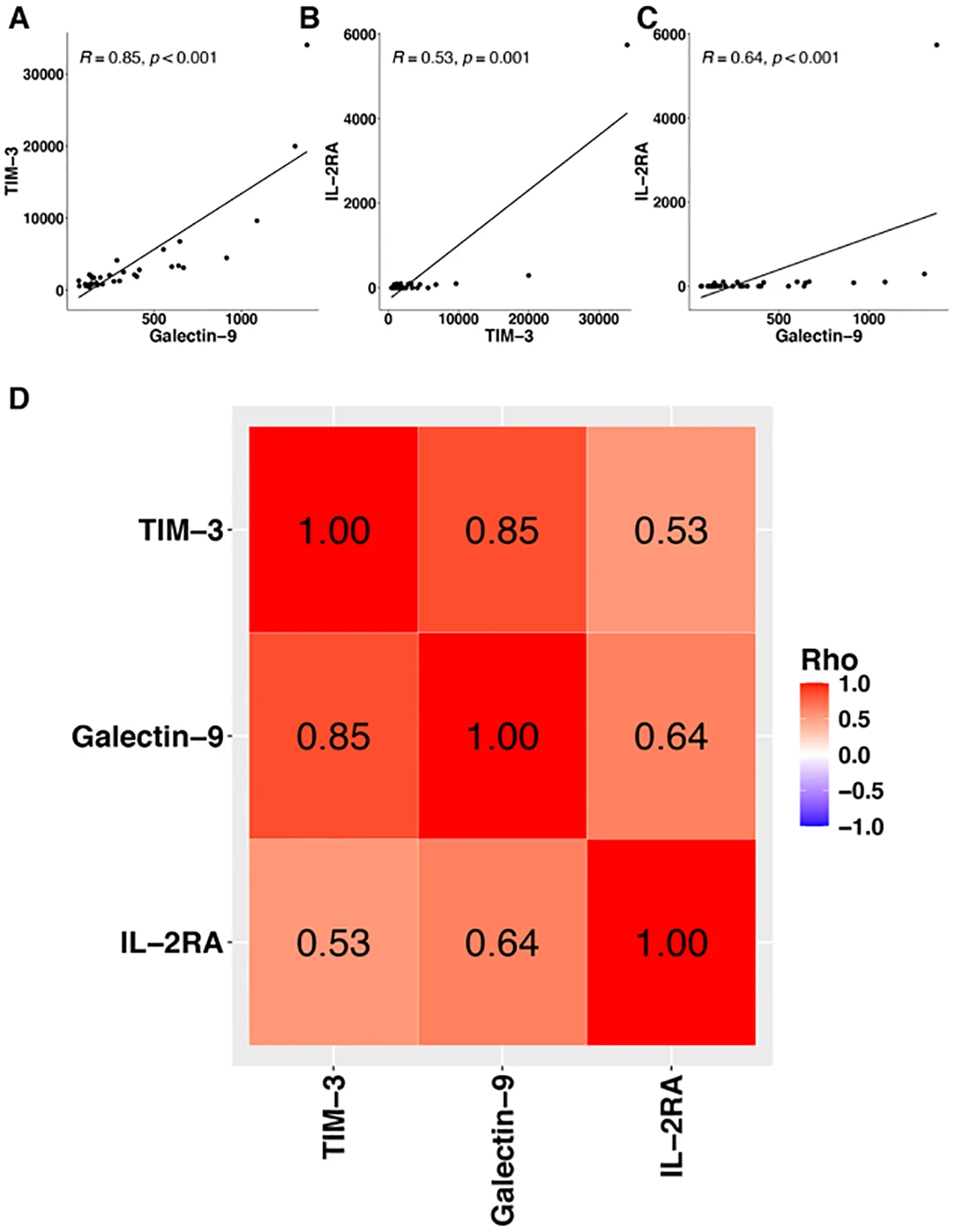
Correlations and clustering of prognostic markers in NMOSD. Scatter plots and correlation analyses showed correlations between TIM-3 and galectin-9 **(A)**, TIM-3 and IL-2Rα **(B)**, and galectin-9 and IL-2Rα **(C)**. Spearman’s rank correlation coefficients (ρ) and exact two-sided p values were as follows: TIM-3 versus galectin-9, ρ=0.85, p<0.001; TIM-3 versus IL-2Rα, ρ=0.53, p=0.001; galectin-9 versus IL-2Rα, ρ=0.64, p<0.001. **(D)** Correlation matrix showed Spearman’s rank correlation coefficients between CSF prognostic markers in NMOSD patients. CSF, cerebrospinal fluid; IL-2Rα, interleukin-2 receptor chain alpha; NMOSD, neuromyelitis optica spectrum disorders; TIM-3, T-cell immunoglobulin and mucin domain 3.

## Discussion

This retrospective single-center study investigated CSF prognostic markers for neuroimmunological diseases using a bead-based multiplex immune-assay. CSF TIM-3 and galectin-9 were significantly elevated in severe NMOSD.

We newly identified elevated CSF levels of TIM-3 and galectin-9 in severe NMOSD patients. It has been reported that TIM-3 tended to be highly expressed in peripheral blood mononuclear cells from NMOSD patients (4). It seems plausible that TIM-3/galectin-9 in CSF serve as a new biomarker associated with disease severity in NMOSD.

Previous studies have reported associations between serum soluble PD-1/PD-L1 and disease status or future relapse in AQP4-IgG-positive NMOSD (3), while peripheral blood LAG-3 expression has been associated with disease severity (4). These findings support the involvement of immune checkpoint pathways in NMOSD. CSF measurements may provide complementary information by sampling the CNS compartment more directly; however, CSF concentrations cannot by themselves distinguish intrathecal production from passive transfer associated with blood-CSF barrier disruption. Paired serum-CSF studies will therefore be required to determine the incremental value of CSF biomarkers over systemic measurements.

Potential confounding by baseline clinical severity should also be considered when interpreting these findings. Patients with severe disability at 6 months had substantially higher pretreatment EDSS than those with mild disability, indicating that the severity of the initial attack may have contributed to subsequent disability. Because only eight patients had severe disability at 6 months, we did not perform multivariable regression analysis, as adjustment for multiple clinical and biomarker variables would likely result in an unstable model and overfitting. The ROC estimates obtained from such a small number of outcome events may be unstable and potentially subject to overestimation. The reported AUCs should be interpreted as exploratory estimates rather than definitive measures of prognostic discrimination. Therefore, the present study cannot establish that CSF TIM-3 or galectin-9 provides independent prognostic information beyond baseline disability and clinical characteristic. Importantly, our study does not establish whether adding CSF TIM-3 or galectin-9 to conventional clinical variables, particularly pretreatment EDSS, improves prognostic discrimination. Because of the small number of outcome events, we were unable to reliably evaluate incremental predictive performance using multivariable models. Future studies with larger cohorts should determine whether these biomarkers provide additional prognostic information beyond established clinical measures. Nevertheless, in the exploratory analysis restricted to patients with pretreatment EDSS ≥ 6, those who improved to EDSS <6 at 6 months had lower CSF TIM-3 and galectin-9 levels than those who remained severely disabled. Although this finding suggests that these biomarkers may provide information beyond baseline disability, the small sample size precludes definitive conclusions regarding their independent prognostic value.

TIM-3 is an immune checkpoint molecule that binds to galectin-9 and contributes to T cells exhaustion, and it is involved in immune regulation in the central nervous system (11). Galectin-9, secreted by activated astrocytes, is known to suppress autoimmune T cells in a TIM-3-dependent manner (12–14), but to enhance cytokine secretion by microglia in a TIM-3-independent manner (15). It is believed that the role of TIM-3/galectin-9 varies depending on cell type, pathological condition, and disease stage, and the function of TIM-3/galectin-9 in the CNS has not yet been fully elucidated. It has been reported that TIM-3 and galectin-9 levels were elevated in the CSF of patients with primary central nervous system lymphoma (PCNSL) (9), and it has been reported that in PCNSL tissue, TIM-3 is highly expressed in tumor cells, tumor-associated macrophages, and lymphocytes, while galectin-9 is highly expressed in tumor-associated macrophages, glial cells, and endothelial cells (16). In another study, TIM-3/galectin-9 was highly expressed in microglia in Alzheimer’s disease model mice (17); therefore, TIM-3/galectin-9 in CSF may not originate from a single cell type but rather serve as a biomarker reflecting glial activation within the CNS. Indeed, activated neutrophils and macrophages, along with T cells, were seen in the tissue destruction associated with the acute phase of NMOSD (18). While TIM-3/galectin-9 signaling acts as a suppressor in T cells, TIM-3 is also expressed on innate immune cells, and it is known that it induces the activation of the innate immune system—such as neutrophil degranulation—via galectin-9 (19). The strong correlation between TIM-3 and galectin-9 suggests that the TIM-3/galectin-9 axis may be associated with innate immune activation and the disease severity of NMOSD. On the other hand, our MOGAD cases showed lower levels of TIM-3 and galectin-9 in the CSF, as well as a mild clinical course. This is consistent with the current understanding of MOGAD as a milder form of disease than NMOSD (20).

Because we measured only soluble TIM-3 and galectin-9 levels in the CSF, we did not assess their cell-surface or membrane-bound expression on immune or other cell populations. Therefore, the present findings do not establish activation of the TIM-3/galectin-9 signaling pathway at the cellular level, and the cellular sources and mechanisms underlying the increased soluble concentrations remain unclear. Elevated CSF levels may reflect intrathecal immune activity, release or shedding from activated cells, or passive transfer associated with disruption of the blood-CSF barrier. Future studies incorporating cellular analyses, including cell-surface expression, together with paired serum-CSF measurements and markers of blood-CSF barrier integrity, will be necessary to clarify the biological significance and origin of these soluble molecules.

IL-2Rα showed moderate prognostic performance and only moderate correlations with TIM-3 and galectin-9. This divergent pattern may reflect differences in the biological processes represented by these molecules. IL-2Rα primarily reflects T-cell activation and proliferation through IL-2 receptor signaling, whereas TIM-3 and galectin-9 constitute a distinct immune checkpoint axis. Therefore, IL-2Rα may provide complementary information regarding inflammatory lymphocyte activation rather than directly reflecting activation of the TIM-3/galectin-9 pathway.

### Limitations and future directions

Several limitations of the present study should be acknowledged. First, this was a retrospective, single-center study with a relatively small sample size, particularly for patients with severe disability at 6 months (n=8). Because of the limited number of outcome events, we did not perform multivariable regression analysis, as adjustment for multiple clinical and biomarker variables would be susceptible to overfitting and unstable estimates. Consequently, we could not determine whether CSF TIM-3 and galectin-9 were independently associated with 6-month disability after adjustment for baseline EDSS, age, sex, CSF protein levels, and other potential confounders. The exploratory analysis restricted to patients with pretreatment EDSS ≥6 suggested that lower TIM-3 and galectin-9 levels were associated with subsequent improvement; however, the very small sample size limits the strength of this observation. Prospective cohorts sufficient sample sizes and appropriate adjustment for baseline clinical variables are needed to validate these findings.

Second, paired serum samples obtained concurrently with CSF sampling were not consistently available in this retrospective cohort. Therefore, we could not directly compare CSF and serum concentrations of the examined biomarkers or determine whether the observed CSF elevations primarily reflected systemic or intrathecal immune responses. Future prospective studies should collect paired serum and CSF samples at standardized time points to clarify the relationship between systemic and CSF biomarker levels.

## Conclusions

In AQP4-IgG-positive NMOSD, higher CSF levels of TIM-3 and galectin-9 during the acute attack phase were associated with severe disability at 6 months. These findings suggest that TIM-3 and galectin-9 may represent candidate biomarkers associated with clinical outcome. However, their independent prognostic value and clinical utility remain uncertain. Large prospective multicenter studies with standardized clinical and paired serum-CSF assessments are required before these biomarkers can be considered for routine clinical use.

## Data Availability

The original contributions presented in the study are included in the article/**Supplementary Material**. Further inquiries can be directed to the corresponding author.
