# Supplementary material for: Soluble TIM-3 and galectin-9 in cerebrospinal fluid are associated with disability at 6 months in aquaporin-4 IgG-positive neuromyelitis optica spectrum disorder
Source: Front Immunol. 2026 Oct 5;17:1923404. doi: 10.3389/fimmu.2026.1923404 (PMC13677341; doi:10.3389/fimmu.2026.1923404)
Supplement: Supplementary file 1 [file DataSheet1.pdf]

Soluble TIM-3 and Galectin-9 in Cerebrospinal Fluid Predict Disability at 6 Months in Aquaporin-4 IgG-Positive Neuromyelitis Optica Spectrum Disorder

Shota Nishii, Ritsu Akatani, Kyoka Shiroma, Ryosuke Takeda, Asato Tsuji, Kimitaka Katanazaka, Shusuke Koto, Norio Chihara*

*Corresponding authors: Norio Chihara
Division of Neurology, Kobe University Graduate School of Medicine, Kobe, Japan.
E-mail: chiharan@med.kobe-u.ac.jp

SUPPLEMENTAL MATERIAL

Supplemental Table 1 Post-hoc pairwise comparisons of demographic and clinical characteristics

Supplemental Table 2 Post-hoc pairwise comparisons of CSF markers between the groups

Supplemental Figure 1 CSF TIM-3 and galectin-9 levels in NMOSD patients with pretreatment EDSS ≥ 6 according to 6-month functional outcome

Supplemental Table 1 Post-hoc pairwise comparisons of demographic and clinical characteristics

Baseline characteristics	Group1	Group2	Adjusted P-value
Age	NMOSD	MOGAD	**0.002**
Age	NMOSD	iNPH	**0.002**
Age	MOGAD	iNPH	**<0.001**
Sex	NMOSD	MOGAD	**0.021**
Sex	NMOSD	iNPH	0.28
Sex	MOGAD	iNPH	0.36
CSF leukocyte	NMOSD	MOGAD	0.28
CSF leukocyte	NMOSD	iNPH	**0.002**
CSF leukocyte	MOGAD	iNPH	**0.035**

Post-hoc pairwise comparisons were performed using Holm-adjusted pairwise Fisher's exact test in the proportions of sex, and Holm-adjusted Dunn's tests in age, CSF leukocyte to compare the groups.
Bold indicates significant difference between groups ($p<0.05$).
CSF, cerebrospinal fluid; iNPH, idiopathic normal pressure hydrocephalus; MOGAD, myelin oligodendrocyte glycoprotein antibody-associated disease; NMOSD, neuromyelitis optica spectrum disorders.

Supplemental Table 2 Post-hoc pairwise comparisons of CSF markers between the groups

Markers	Group1	Group2	Adjusted P-value
TIM-3	Severe NMOSD	Mild NMOSD	**0.011**
TIM-3	Severe NMOSD	MOGAD	**0.006**
TIM-3	Severe NMOSD	iNPH	**<0.001**
TIM-3	MOGAD	iNPH	0.40
Gelectin-9	Severe NMOSD	Mild NMOSD	**0.008**
Gelectin-9	Severe NMOSD	MOGAD	0.14
Gelectin-9	Severe NMOSD	iNPH	**0.002**
Gelectin-9	MOGAD	iNPH	0.34
IL-2Rα	Severe NMOSD	Mild NMOSD	**0.006**
IL-2Rα	Severe NMOSD	MOGAD	**0.099**
IL-2Rα	Severe NMOSD	iNPH	**0.004**
IL-2Rα	MOGAD	iNPH	0.72
LAG-3	Severe NMOSD	Mild NMOSD	**0.035**
LAG-3	Severe NMOSD	MOGAD	**0.045**
LAG-3	Severe NMOSD	iNPH	0.52
LAG-3	MOGAD	iNPH	0.66
B7.2	Severe NMOSD	Mild NMOSD	**0.003**
B7.2	Severe NMOSD	MOGAD	**0.019**
B7.2	Severe NMOSD	iNPH	0.26
B7.2	MOGAD	iNPH	0.38
FasL	Severe NMOSD	Mild NMOSD	0.40
FasL	Severe NMOSD	MOGAD	0.54
FasL	Severe NMOSD	iNPH	**<0.001**
FasL	MOGAD	iNPH	**0.038**

Post-hoc pairwise comparisons were performed using Holm-adjusted Dunn's tests in CSF marker to compare between severe NMOSD and the other groups, as well as between MOGAD and iNPH.
Bold indicates significant difference between groups ($p<0.05$).
CSF, cerebrospinal fluid; FasL, Fas ligand; IL-2Rα,interleukin-2 receptor chain alpha; iNPH, idiopathic normal pressure hydrocephalus; LAG-3, lymphocyte activation gene 3; MOGAD, myelin oligodendrocyte glycoprotein antibody-associated disease; NMOSD, neuromyelitis optica spectrum disorders; TIM-3, T-cell immunoglobulin and mucin domain 3.

Supplemental Figure 1 CSF marker levels in NMOSD patients with pretreatment EDSS ≥ 6 according to 6-month functional outcome

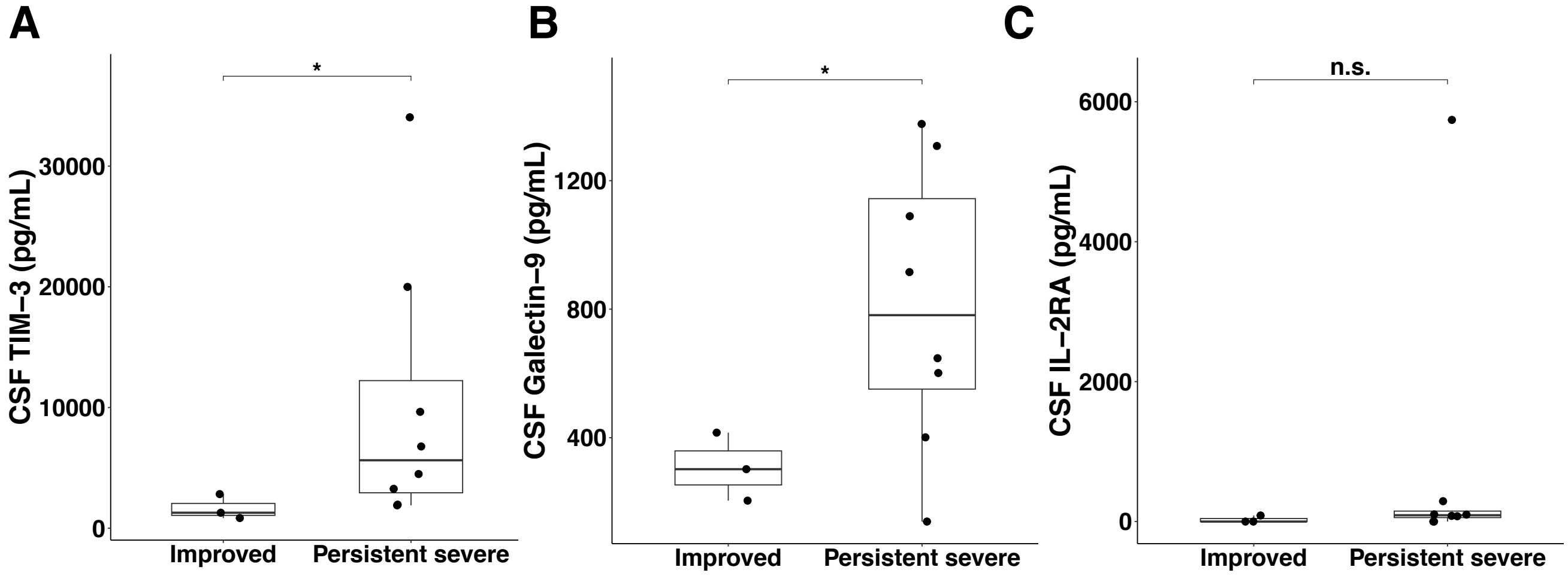

Boxplots depict the distribution of CSF TIM-3 (A), galectin-9 (B), and IL-2Rα (C) in NMOSD patients. Patients with pretreatment EDSS ≥ 6 were stratified according to their functional outcome at 6 months into an improved group (6-month EDSS < 6) and a persistent severe group (6-month EDSS ≥ 6). Median values are represented by the horizontal bar, IQR by boxes, 1.5× IQR by whiskers, and individual values by dots. *, $p < 0.05$. n.s., not significant. Brunner-Munzel's tests were used to compare between improved and persistent severe NMOSD.

CSF, cerebrospinal fluid; IL-2Rα,interleukin-2 receptor chain alpha; IQR, interquartile range; NMOSD, neuromyelitis optica spectrum disorders; TIM-3, T-cell immunoglobulin and mucin domain 3.

.
